# Myocardial Bridging as a Typical Cause of Chest Pain in a Psychiatric Patient: A Case Report

**DOI:** 10.7759/cureus.87232

**Published:** 2025-07-03

**Authors:** Tatiana Oliveira

**Affiliations:** 1 Family Medicine, Unidade de Saúde Familiar (USF) Renascer, Unidade Local de Saúde (ULS) Santo António, Porto, PRT

**Keywords:** angina, anxiety, diagnostic challenge, ischemia, myocardial bridge, non-obstructive coronary disease

## Abstract

Chest pain is a frequent cause of emergency medical visits and can result from either benign or life-threatening conditions. While atherosclerotic coronary artery disease is the most common etiology, other causes of myocardial ischemia, such as congenital anomalies like myocardial bridging, should also be considered.

This case report is about a 45-year-old male with a psychiatric history of major depressive disorder and generalized anxiety, under treatment with fluvoxamine. He had multiple visits over six years to primary care for chest pain episodes consistently attributed to anxiety, with normal ECG findings. In 2024, he presented with worsening chest pain at rest, fatigue, and exertional dyspnea. While initial assessment in the emergency department was unremarkable, further evaluation showed ischemia on stress testing and hypokinesis on echocardiogram. Coronary angiography revealed a myocardial bridge in the left anterior descending artery with no obstructive lesions. He was discharged on beta-blockers and referred for outpatient cardiology follow-up.

This case highlights the diagnostic challenge of chest pain in the presence of psychiatric comorbidities. It underlines the importance of comprehensive evaluation and consideration of less common causes of ischemia, such as myocardial bridging, especially when symptoms persist despite reassuring initial tests.

Myocardial bridging, although frequently asymptomatic, may present with angina-like symptoms and should be part of the differential diagnosis for non-obstructive ischemia. A careful and holistic clinical approach is essential to avoid diagnostic delays, particularly in patients with overlapping psychiatric symptoms. Furthermore, this case aims to raise awareness among physicians about less common causes of chest pain.

## Introduction

Chest pain is one of the most frequent reasons for seeking medical care in emergency settings. Distinguishing between benign causes and potentially fatal conditions, such as ischemic heart disease, is imperative.

Among the benign causes, anxiety and depressive disorders are common contributors. They can produce chest pain through autonomic nervous system activation and hyperventilation, leading to symptoms without an underlying cardiac cause. This type of pain is usually prolonged, poorly localized, and occurs at rest, in contrast to typical ischemic pain, which is triggered by physical exertion, has a pressing character and short duration, and is relieved by rest or nitrates. Clinical distinction between these entities is essential, although they often coexist and complicate the diagnostic process [1].

While atherosclerosis remains the primary etiology of ischemic chest pain, clinicians must also recognize less common but potentially serious causes such as coronary vasospasm and congenital anomalies like myocardial bridging [2,3]. Myocardial bridges are segments of coronary arteries, most often the left anterior descending artery, that course intramyocardially rather than following the typical epicardial route [2,4]. Though frequently asymptomatic, myocardial bridge can be associated with myocardial ischemia, arrhythmias, or even sudden cardiac death in rare cases [4,5]. Diagnosis is typically made by coronary angiography [4]. Medical treatment is preferred in cases of symptomatic or documented ischemia, with beta-blockers or ivabradine as first-line therapies due to their effects on heart rate and contractility [6]. Calcium channel blockers may also be considered. However, nitrates are contraindicated as they may increase contractility [5]. Surgical intervention is reserved for refractory cases [4]. This case report aims to raise awareness of a less common etiology of chest pain and highlight the diagnostic and clinical management challenges.

## Case presentation

A 45-year-old male logistics coordinator, with a medical history of major depressive disorder and generalized anxiety, without cardiovascular risk factors and medicated with fluvoxamine, had been regularly monitored in primary care since 2018 due to worsening of his pathology in the context of mourning the death of his mother.

In 2024, he presented again with a one-week history of squeezing chest pain, present at rest. An electrocardiogram performed the previous month was normal, so anxiety was again suspected, and he was referred for stress testing and transthoracic echocardiography. Given his previous similar episodes and psychiatric history, the symptoms were initially attributed to anxiety. However, no emotional trigger was identified, and the persistence of symptoms suggested the need for further evaluation.

Seven days later, still without having performed the prescribed tests because he thought it would be a self-limiting episode again, he returned with worsening chest pain, classified as 9/10, non-radiating, and present both at rest and during exertion. This time, it was associated with fatigue and dyspnea with minimal to moderate effort. These new features raised concern for a cardiac cause.

**Figure 1:**
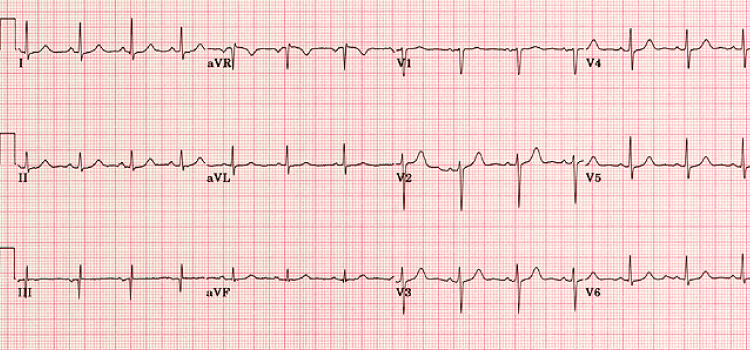
Electrocardiogram. Normal electrocardiogram during an angina episode.

He was referred to the emergency department, where physical exam, electrocardiogram (Figure 1), and cardiac biomarkers (troponins) were unremarkable.

**Figure 2:**
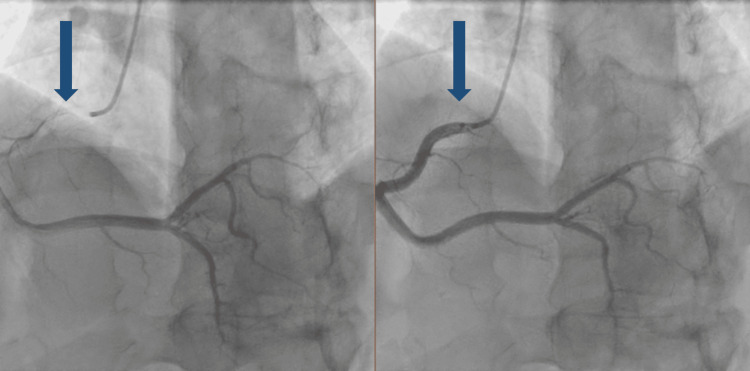
Coronary angiography. During left ventricular systole, the intramuscular segment of the vessel is compressed, a condition referred to as "milking".

Following discharge, he completed the prescribed exams. The stress test was clinically and electrically positive for ischemia, and the transthoracic echocardiography showed mildly reduced ejection fraction (48%) with hypokinesis of the anterior and lateral walls. He was hospitalized for coronary angiography, which revealed a myocardial bridge in the left anterior descending artery and non-obstructive irregularities in other coronary arteries (Figure 2). He was discharged with beta-blocker therapy and was referred for a cardiology consultation, from which he was discharged, since he remained asymptomatic with the medication and, therefore, did not require follow-up.

## Discussion

This case illustrates the complexity of identifying the etiology of chest pain in patients with no evidence of obstructive coronary artery disease on initial investigations. Anxiety and depression can cause non-cardiac chest pain, typically prolonged, poorly localized, and occurring at rest. In contrast, ischemic pain is brief, pressure-like, and triggered by exertion. Distinguishing between them is essential, though they often coexist and complicate diagnosis [1]. There is a strong bidirectional link between coronary artery disease and psychiatric disorders. Patients with severe mental illness have a higher risk of developing coronary artery disease, with prevalence reaching 9.9%. Conversely, depression and anxiety are common in coronary artery disease patients, affecting approximately 20% and 23%, respectively. These data highlight the need for integrated cardiovascular and mental health care [7,8].

While atherosclerosis remains the most frequent cause of myocardial ischemia, other entities such as congenital coronary anomalies, particularly myocardial bridging, must be considered. A myocardial bridge is a rare condition in which a segment of a coronary artery, most commonly the left anterior descending artery, tunnels through the myocardium instead of following the normal epicardial course. Its prevalence is estimated at 1.7% in angiographic studies but can reach up to 25% in autopsy series [2,4]. Although usually asymptomatic, myocardial bridge can be associated with chest pain due to systolic compression of the artery, and in rare cases, may lead to arrhythmias or even sudden cardiac death [4,6]. Chest pain can typically occur in deeper or longer myocardial bridges [4,6]. Coronary angiography remains the gold standard for diagnosis [2]. Medical treatment is recommended when symptoms or ischemia are documented. First-line therapy includes beta-blockers or ivabradine, which reduce heart rate and myocardial contractility [2,4]. Non-dihydropyridine calcium channel blockers may also be effective [4,6]. Importantly, nitrates are contraindicated as they may exacerbate symptoms by increasing contractility [5]. Surgical intervention is reserved for refractory cases unresponsive to medical management [2,5].

## Conclusions

This case highlights the diagnostic challenges posed by psychiatric comorbidities, such as generalized anxiety disorder, which may bias clinical judgment and delay appropriate investigation. In patients with persistent symptoms, especially those labeled as anxiety-related, clinicians must remain vigilant and pursue further diagnostic workup when warranted. It also emphasizes the relevance of investigating persistent symptoms even in the absence of abnormal initial findings. Family physicians should be aware of atypical causes of chest pain, such as myocardial bridging, to ensure early recognition and intervention.
